# Different MRI Signs in Predicting the Treatment Efficacy of Epidural Blood Patch in Spontaneous Intracranial Hypotension: A Case Report

**DOI:** 10.5812/iranjradiol.3929

**Published:** 2013-08-30

**Authors:** Ching Wen Huang, Yuh Feng Tsai, Chen Yu Hsiao

**Affiliations:** 1 Department of Diagnostic Radiology, Shin-Kong Wu Ho-Su Memorial Hospital, Taipei, Taiwan; 2 College of Medicine, Fu-Jen Catholic University, New Taipei City, Taiwan; 3 Neuroimaging Center, Department of Health Management, Shin-Kong Wu Ho-Su Memorial Hospital, Taipei,Taiwan

**Keywords:** Blood Patch, Epidural, Pituitary Gland, Posterior, Intracranial Hypotension, Magnetic Resonance Imaging, Cranial Sinuses

## Abstract

Abstract

The current mainstay of treatment in spontaneous intracranial hypotension (SIH) is an epidural blood patch (EBP). Although magnetic resonance imaging (MRI) has a well-established role in the diagnosis of SIH, imaging features regarding the treatment efficacy of EBP have rarely been discussed. We therefore sought to investigate and compare the sequential brain MRI studies before and after EBP by evaluating the changes of the following intracranial structures—the contour of the transverse dural sinus (TDS), tension of the pituitary stalk (or the infundibulum), and thickness of the dura mater. We found that the progressive reversals of these structures are predictive of an effective EBP.

## 1. Introduction

Spontaneous intracranial hypotension (SIH) is caused by leakage of the cerebrospinal fluid (CSF) from a tear in the dura mater, with an estimated incidence of 5 per 100,000 annually (1, 2). SIH has been increasingly diagnosed since the application of MRI with the most typical imaging findings consisting of dural thickening, pachymeningeal enhancement, engorgement of venous structures, subdural effusion or collections, pituitary hyperemia and brain sagging (1). The prototypical symptom is an orthostatic headache that occurs or worsens with the upright posture (3-5) because of brain sagging with traction of the pain-sensitive dura or compensatory dilatation of the pain-sensitive venous structures (1). Among the associated symptoms, neck stiffness, nausea and vomiting are due to meningeal irritation while diplopia, cranial neuropathies and hearing impairment are the results of brain sagging with stretching of the nerve complexes (1). A definite diagnosis can be made when the CSF opening pressure is less than 60 mm H_2_O at a lumbar puncture (1, 6, 7). The current mainstay of treatment is epidural blood patch (EBP) that forms a dural tamponade to seal CSF leaks by injection of the autologous blood into the spinal epidural space (1). EBP is also effective in relieving symptoms instantaneously in about one third of the patients and thus it may serve a diagnostic purpose (1, 8, 9). Recently, some authors have proposed the use of heavily T2-weighted MR myelography (MRM) for both the diagnosis and follow-up purposes in SIH (10). In this article, we sought to find some indicators on brain MRI to evaluate the treatment efficacy of EBP, without the need for special MR pulse sequences. The use of these indicators allows accurate prediction of EBP efficacy and helps physicians determine the next treatment step.

## 2. Case Presentation

**Figure 1.**
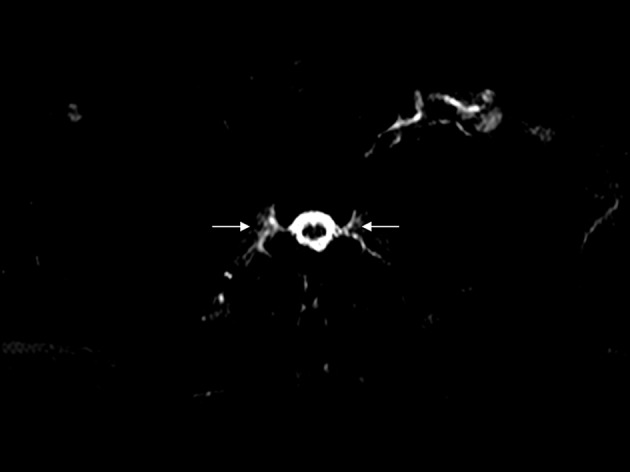
Spinal MR myelography T3 of the thoracic spine; axial heavily T2- weighted image, TE=750 ms demonstrates abnormal fluid accumulation along bilateral nerve sleeves, suggestive of CSF leaks (arrows).

A 65-year-old man suffered from right fronto-parietal headache on and off and right neck soreness for seven months. The headache, by his description, was not related to posture. Empirical analgesics were given about 3 months prior to admission, but the headache did not subside. Few days before admission, he developed orthostatic headache and spontaneous intracranial hypotension (SIH) was suspected. Brain and spinal MRI studies were performed and SIH was diagnosed. Since MR myelography had depicted CSF leaks bilaterally along the epidural space from T1 to T6 (Figure 1, T3 level demonstrated), epidural blood patch (EBP) was performed with the injection of autologous blood (30 cc) into T6 epidural space and a trendelenburg position for 2 hours after the procedure to allow ascending of blood over several segments. 

**Figure 2.**
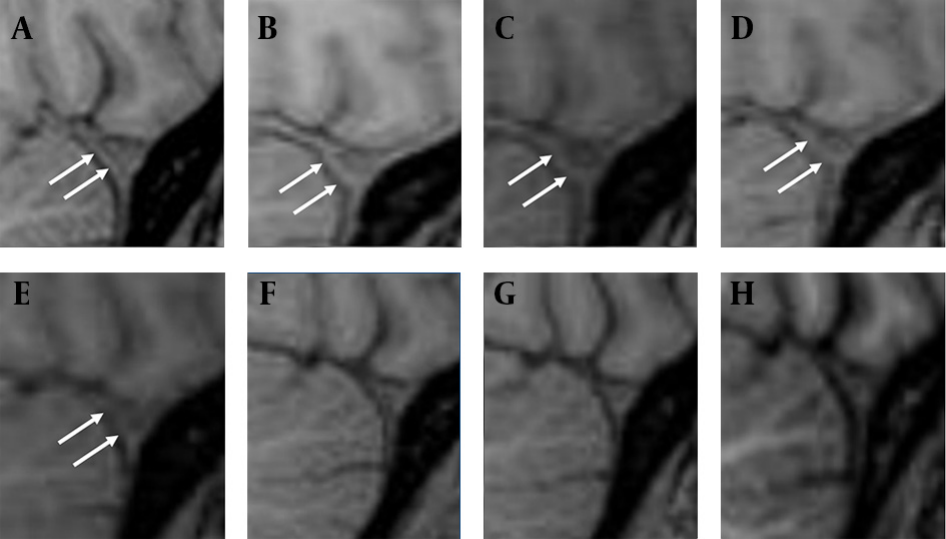
Sequential imaging changes of the contour of the transverse dural sinus (TDS) on the precontrast sagittal T1W imaging. A and B, two months and three days before EBP; C, the first day after EBP; D-H ten days, half a month, one month, two months and three months after EBP. Normally, the anterior border of TDS should be linear or concave in shape. Bulging or convex shape, i.e., “venous engorgement” sign, suggests intracranial hypotension. The contour of TDS is normal two months before EBP on A (arrows), and bulged three days before EBP on B (arrows) and the first day after EBP on C (arrows). There is mild reversal of the anterior convexity of TDS ten days after EBP on D (arrows). The reversal completes by half a month after EBP on E (arrows) and quite stationary thereafter on F-H.

**Figure 3.**
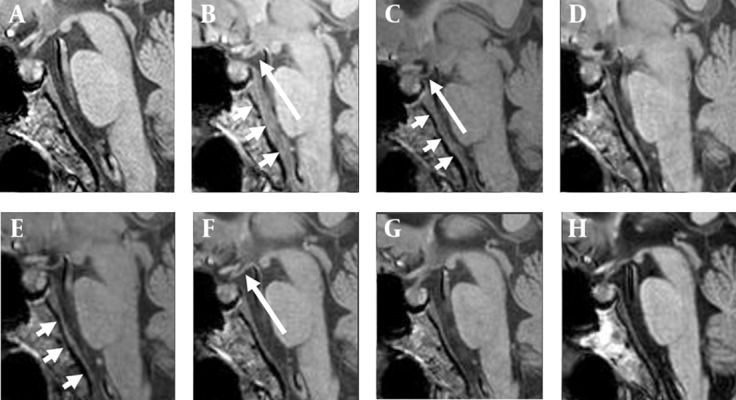
Sequential imaging changes of the infundibulum tension and dural thickness on the sagittal T1W imaging. A and B, two months and three days before EBP; C, the first day after EBP; D-H, ten days, half a month, one month, two months and three months after EBP. Dural thickening is obvious three days before EBP on B (short arrows) and the first day after EBP on C (short arrows). There is a significant regression of dural thickening by half a month after EBP on E (short arrows). Mild “infundibulum laxity” is found three days before EBP on B (long arrow), but it is still hard to denote “brain sagging”. It becomes obvious on the day of EBP on C (long arrow). Nearly complete reversal of the infundibulum tension is shown by one month after EBP on F (long arrow).

Eight brain MRI studies were reviewed and compared collectively, among which two were performed two months and three days before EBP, and the others were performed on the first day, 10th day, half a month, one month, two months and three months after EBP. The tension of the infundibulum, contour of the transverse dural sinus (TDS) and dural thickness of the clivus were evaluated on sagittal T1-weighted imaging. The contour of the engorged TDS (Figure 2) and dural thickening (Figure 3) returned to normal half a month after EBP, whereas the tension of the infundibulum became normal one month after EBP (Figure 3). 

## 3. Discussion

To seal the leak in the spine in SIH, the mainstay of current treatment is an EBP. In addition, repeated EBP procedures or even surgical intervention may be required if the initial attempt fails (1). It is therefore crucial to evaluate the treatment efficacy of each EBP, for it helps physicians determine whether another procedure should be prompted, especially for those who do not get instantaneous symptom relief after EBP.

Imaging studies such as conventional myelography and computed tomographic myelography are capable of depicting spinal CSF leaks (11). However, the need for lumbar punctures in such procedures may worsen the underlying CSF leaks. In contrast, MR myelography (MRM), taking the advantage of its noninvasiveness, has been reported to be effective in detecting spinal CSF leaks and is useful for the diagnostic and follow-up purposes in SIH (10). In our experiences, the interpretation of spinal CSF leaks on MRM is not an easy task and relies on the reviewer’s expertise. From these points of view, other noninvasive and more reviewer-friendly imaging techniques are crucial to provide information regarding the treatment efficacy of EBP.

Typical imaging features in SIH include dural thickening, pachymeningeal enhancement, engorgement of the intracranial vessels, subdural effusion, pituitary hyperemia and brain sagging. These abnormal findings are the results of spinal CSF leaks. After an effective EBP, the intracranial pressure would restore and these abnormal intracranial structures are supposed to return to normal after time. As demonstrated in our case, the “reversal of transverse dural sinus (TDS)” and “reversal of dural thickness” take half a month to complete, and the “reversal of infundibulum tension” takes a relatively longer time, a month, to complete after a successful EBP. These imaging changes can be identified directly on the routine brain MRI without needing a special imaging technique.

Literature regarding the prediction of successful treatment in SIH by evaluating the intracranial structures is scanty. Farb et al. (12) who first used the term “venous distention sign” (VDS) to describe the engorgement of the TDS in intracranial hypotension, suggested VDS was the first sign to disappear after successful treatment, in comparison with the regression of pachymeningeal enhancement and the pituitary gland size (12, 13). In our study, a similar time sequence regarding the reversals of these structures was observed. However, their results are based on four cases, each undergoing only one short-term MRI study (2, 4, 7 and 16 days), but ours are based on a series of short-term MRI studies that can offer a better chronological demonstration of the imaging changes. When compared with their study, there are apparent variations in the reversal time of the TDS after treatment, which may be related to the individual difference in disease chronicity and severity. Measuring the “venous hinge” angle (VHA), i.e. the angles between the vein of Galen and the internal cerebral vein, has also been proposed to be used in evaluating the treatment efficacy (14). The VHA is decreased in intracranial hypotension and will return to the baseline after treatment (14). We think the decrease in VHA reflects the degree of brain sagging, a mechanism similar to “infundibulum laxity” as will be discussed later in this article.

The definite mechanism why these abnormal structures do not reverse simultaneously is unclear but reasonable. As we know, the main cause of venous engorgement in SIH is intracranial hypotension. Once the intracranial pressure restores, the engorged vessels respond immediately that account for the earlier reversal of an engorged vein after EBP. With a likewise mechanism, dural thickening in SIH is due to hydrostatic change in the CSF that in turn leads to “venous engorgement” in the dura mater ( 1 , 12 , 13 ). Therefore, earlier reversal of dural thickening can be expected as that of an engorged dural vein. As for the reversal of the infundibular tension, we think it is more relevant to the re-establishment of prepontine CSF volume than the restoration of the intracranial pressure, and therefore more time consuming (Figure 2). 

Brain sagging, resulted from CSF volume depletion, is one of the typical MRI features in SIH. The loss of brain buoyancy also leads to “drooping” of the pituitary stalk, hence the name of “infundibulum laxity” sign in this article. Here, we put forth the infundibulum laxity sign in the diagnosis of SIH, for it is a more direct and apparent sign. In contrast, the interpretation of brain sagging is not always easy on MRI, especially when it is subtle.

In summary, the “infundibulum laxity sign” is a more obvious MRI sign and aids in the diagnosis of SIH. The reversals of the following structures, including the contour of the transverse sinus, the infundibulum tension and the dural thickness, are useful and reviewer-friendly MRI signs in predicting EBP efficacy. However, these MRI signs are findings from just one case, and more case reports or researches on this issue are required to evaluate the real efficacy of EBP on pre- and post-EBP MR imaging changes correlated with clinical presentations.
